# Mithramycin downregulates proinflammatory cytokine-induced matrix metalloproteinase gene expression in articular chondrocytes

**DOI:** 10.1186/ar1735

**Published:** 2005-04-04

**Authors:** Abdelhamid Liacini, Judith Sylvester, Wen Qing Li, Muhammad Zafarullah

**Affiliations:** 1 Département de Médecine and Centre de Recherche du Centre Hospitalier de l'Université de Montréal (CHUM), Hôpital Notre-Dame du CHUM, Montréal, Québec, Canada

## Abstract

Interleukin-1 (IL-1), IL-17 and tumor necrosis factor alpha (TNF-α) are the main proinflammatory cytokines implicated in cartilage breakdown by matrix metalloproteinase (MMPs) in arthritic joints. We studied the impact of an anti-neoplastic antibiotic, mithramycin, on the induction of MMPs in chondrocytes. MMP-3 and MMP-13 gene expression induced by IL-1β, TNF-α and IL-17 was downregulated by mithramycin in human chondrosarcoma SW1353 cells and in primary human and bovine femoral head chondrocytes. Constitutive and IL-1-stimulated MMP-13 levels in bovine and human cartilage explants were also suppressed. Mithramycin did not significantly affect the phosphorylation of the mitogen-activated protein kinases, extracellular signal-regulated kinase, p38 and c-Jun N-terminal kinase. Despite effective inhibition of MMP expression by mithramycin and its potential to reduce cartilage degeneration, the agent might work through multiple unidentified mechanisms.

## Introduction

A major pathological manifestation of patients with osteoarthritis (OA) and rheumatoid arthritis is the degeneration of articular cartilage [1,2]. Matrix metalloproteinases (MMPs) such as MMP-3 and MMP-13 are known to cleave collagens and aggrecan of cartilage extracellular matrix [3-5]. The concentrations of several MMPs are increased in cartilage, synovial membrane and synovial fluid of patients with arthritis [6,7]. Indeed, cartilage-specific overexpression of active human MMP-13 causes OA in mice [8]. Proinflammatory cytokines, interleukin-1 (IL-1), IL-17 and tumor necrosis factor (TNF)-α are also increased in arthritic joints and are known to induce catabolic pathways leading to an enhanced expression of MMPs [9-11]. Inhibition of these proteases is regarded as an important approach for reducing damage in arthritic tissues [12].

AP-1 binding sites found in the promoter regions of the genes encoding MMP-3 and MMP-13 are essential for the expression of these genes [13,14]. Sp1 transcription factor is a zinc-finger type transcription factor whose binding sites are found in numerous housekeeping and inducible genes [15]. Human MMP-13 promoter has one putative Sp1 consensus site [16]. Mithramycin is an aureolic acid anti-neoplastic antibiotic that is used for treating cancer-related hypercalcemia [17]. Previous work has revealed that it inhibits bone resorption *in vitro*, possibly by interfering with bone cell lysosomal enzymes [18]. It also prevents the binding of Sp1 transcription factor to its cognate site in DNA by modifying the CG sequences [19]. Here we have studied the impact of mithramycin on proinflammatory cytokine-induced MMP expression. We show for the first time that mithramycin potently suppresses MMP induction by IL-1, IL-17 and TNF-α in chondrocytic cells without impairing the activation of mitogen-activated protein kinases (MAPKs).

## Materials and methods

### Primary cultures of human and bovine chondrocytes, SW1353 cells and treatments

Human cartilage was acquired from the femoral heads of OA patients who underwent hip-replacement surgery at the Notre-Dame Hospital. Normal bovine cartilage was obtained from the knee and hip joints of adult animals from a local abattoir. Chondrocytes were released by 90 min pronase and 9 hours digestion with collagenase (Sigma type IA). The cells were washed with PBS and grown in DMEM containing 10% FCS as high-density primary monolayer cultures until confluent growth. Cells were distributed in six-well plates, grown to confluence, washed with PBS and kept in serum-free DMEM for 24 hours; mithramycin (from Sigma-Aldrich Canada Ltd, Oakville, Ontario; dissolved in water as a 10 mM solution) was then added without medium change at final concentrations of 100 and 150 nM (doses known to inhibit Sp1 binding [20]) for 30 min before treatment for 24 hours with human recombinant IL-1β (10 ng/ml), TNF-α (20 ng/ml) and IL-17 (20 ng/ml) (R&D systems, Minneapolis, MN). The human chondrosarcoma cell line SW1353 was obtained from the American Type Culture Collection (ATCC, Manassas, VA) and treated as described for primary chondrocytes.

### Northern hybridization analysis

Total cellular RNA was extracted by the guanidinium procedure [21] and aliquots of 3 to 5 μg were analyzed by electrophoretic fractionation in 1.2% formaldehyde-agarose gels. The integrity and quantity of RNA were verified by ethidium bromide staining of the 28S and 18S ribosomal RNA bands. The RNA was transferred onto Zeta-probe nylon membrane with a Bio-Rad Transblot in the presence of 0.5 × TAE (Tris-acetate-EDTA) buffer at a current of 500 mA for 12 hours. Northern blots were hybridized with a human stromelysin cDNA probe generously provided by Dr Richard Breathnach (Nantes, France). This probe was a 1.6-kilobase *Eco*RI cDNA fragment cloned in the plasmid pGEM-4Z (Promega Biotech, Madison, WI) and the vector was linearized with *Nar*I. A 491-base-pair RT-PCR-generated [22] and cloned collagenase-3 cDNA was linearized with *Eco*RI. The human 28S ribosomal RNA plasmid (ATCC) was digested with *Xba*I. All antisense RNA probes were synthesized with T7 polymerase in accordance with the protocols of Promega Biotech and were labeled to high specific radioactivity (10^8 ^c.p.m./μg) with [α-^32^P]CTP (3,000 Ci/mmol; Perkin Elmer Life Sciences Inc., Boston, MA).

### Western immunoblot analysis

Total secreted proteins from the 2 to 3 ml of conditioned medium of the chondrocytes or SW1353 cells were concentrated by precipitation with trichloroacetic acid, quantified with the Bio-Rad protein assay system and different amounts of protein aliquots adjusted to 15 μl with 4 × sample buffer comprising 62.5 mM Tris-HCl, 20% glycerol, 0.032% bromophenol blue, 5% mercaptoethanol and 2% SDS. Along with the prestained broad-range molecular mass standards (Bio-Rad), samples were fractionated by a 4% stacking and 10% SDS-PAGE mini gel (Bio-Rad, Mississauga, ON) and transferred to nitrocellulose membrane by electroblotting at 200 mA in a buffer comprising 25 mM Tris-HCl, 192 mM glycine, 0.04% SDS and 20% ethanol. The membranes were rinsed with distilled water, incubated for 1 hour in PBS pH 7.4 with 5% Carnation non-fat milk to block non-specific interactions, and washed five times (twice for 5 min, once for 15 min and twice for 5 min) with PBS containing 0.1% Tween. They were then reacted overnight sequentially in the same buffer at 4°C with 1 to 2 μg/ml anti-human MMP-3 (developed in mouse) and MMP-13 (hinge region, developed in rabbit) antibodies (from Sigma-Aldrich). Subsequently, membranes were washed at 22°C five times with PBS containing 0.1% Tween, incubated with the anti-rabbit or anti-mouse secondary peroxidase-conjugated IgG (300 mU/ml), and washed seven times with PBS containing 0.1% Tween. To reveal the MMP-3 and MMP-13 bands, membranes were incubated with 10 μl of solution A and 990 μl of solution B of the chemiluminescence detection system of Roche Biochemicals (Laval, Québec) and exposed to film for 2 to 15 min.

For Western blots of MAPKs, human femoral-head chondrocytes were pretreated with mithramycin for 30 min and then stimulated with IL-1β for 20 min; total cellular protein extracts (20 μg) in lysis buffer (62.5 mM Tris-HCl pH 6.8, 10% glycerol, 1% Triton X-100, 50 mM dithiothreitol, 2% SDS, 0.01% bromophenol blue) were resolved by 10% SDS-PAGE, transferred to nitrocellulose membranes by electroblotting and incubated overnight at 4°C with primary phosphorylation-state-specific antibodies for phosphorylated extracellular signal-regulated kinase (p-ERK), phospho-p38 and phosphorylated c-Jun N-terminal kinase (p-JNK) (from Cell Signaling Technology Inc., Beverley, MA) at 1:1,000 dilution in 5% BSA, 1 × Tris-buffered saline and 0.1% Tween. Proteins were detected with the enhanced chemiluminescence system from Pharmacia-Amersham. Subsequently, the membranes were stripped with a buffer (containing 100 mM 2-mercaptoethanl, 2% SDS and 62.5 mM Tris-HCl, pH 6.8) at 55°C and reprobed with the antibodies detecting total ERK, p38 and JNK.

### Cartilage explants

Human or bovine femoral head cartilage explants were maintained for 1 week in DMEM with 10% FCS, medium was then changed with 0.01% serum-containing DMEM for 3 days until treatments. Explants were treated with mithramycin and IL-1 vehicles as control (water and PBS-0.1% BSA) or exposed to mithramycin (150 nM) and IL-1 (33 ng/ml) for 15 days with replacement of the fresh reagents every 2 days; the secreted media were concentrated by precipitation with 10% trichloroacetic acid and equal amounts of protein (16 μg per lane for human explants and 20 μg per lane for bovine explants) were subjected to Western immunoblotting as described above. All the experiments described in this paper were performed at least two (primary human chondrocytes and cartilage) or three (SW1353 and bovine chondrocytes) times and the results were reproducible.

## Results

### Mithramycin blocks IL1-stimulated expression of MMP-3 and MMP-13 in human and bovine chondrocyte cell lines

**Figure 1:**
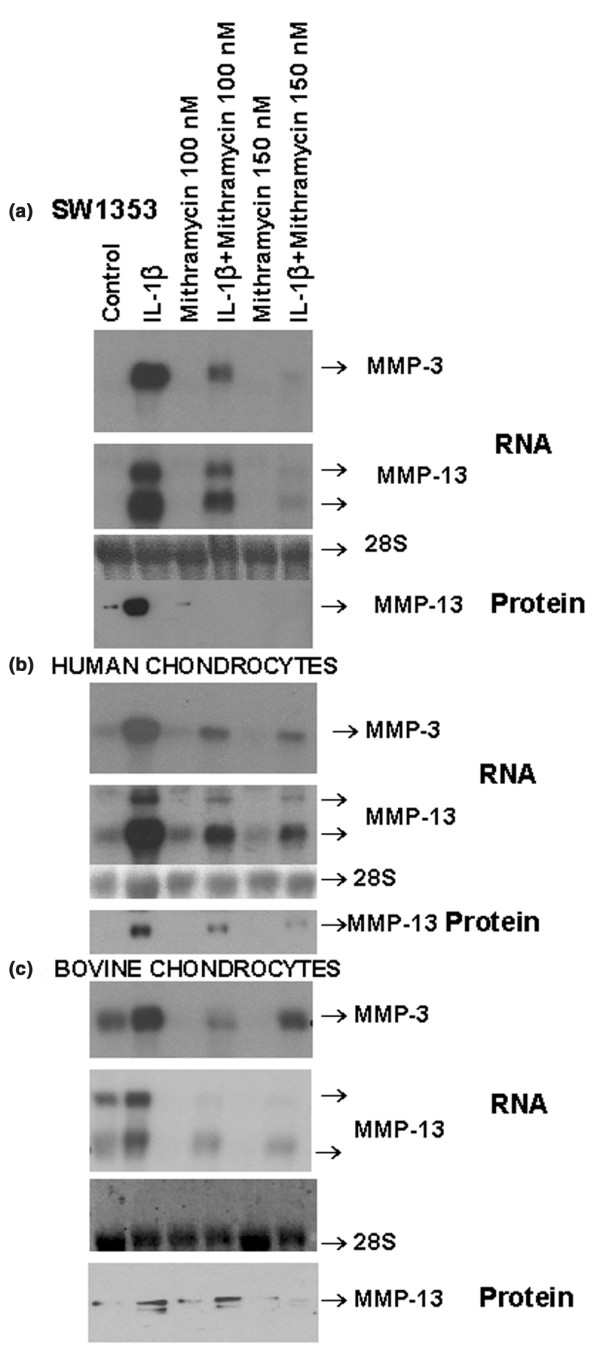
Repression of interleukin (IL)-1β-inducible matrix metalloproteinase (MMP)-3 and MMP-13 RNA expression by mithramycin. Quiescent human chondrosarcoma **(a)**, primary human chondrocytes **(b) **or bovine chondrocytes **(c) **were pretreated with different concentrations of mithramycin for 30 min, followed by additional treatment with IL-1β for 24 hours. The MMP-3, MMP-13 and 28S RNA levels were measured by Northern hybridization, and MMP-13 protein levels were measured by Western blot analysis. For protein gels, 3 μg **(a) **or 4 μg **(b, c) **of protein was applied to each lane. The resulting autoradiograms indicating the respective gene products are shown.

IL-1β potently induced expression of the genes encoding MMP-3 and MMP-13 in the human chondrocytic cell line SW1353 and in primary human femoral head chondrocytes. Mithramycin, a hypocalcemic antibiotic, potently blocked the induction of MMP-3 and MMP-13 mRNA by IL-1β without affecting the control 28S rRNA levels (Fig. 1a,b). The two MMP-13 mRNA bands correspond to transcripts produced by differential use of polyadenylation sites at the 3' end of the gene, the upper band being the longest transcript as reported previously [23]. Induction of MMP-13 protein was also similarly inhibited (Fig. 1a,b). To examine whether mithramycin could also affect MMP gene expression in articular chondrocytes from other species, adult bovine chondrocytes (an important model system in cartilage research) were exposed to different concentrations of mithramycin and then stimulated with IL-1β. This cytokine induced MMP-3 and MMP-13 mRNA expression above basal levels, and pretreatment with mithramycin reduced both constitutive and induced expression in a fashion similar to that of human cells (Fig. 1c). The induction of MMP-13 protein (the main collagen-degrading MMP) by IL-1 was also inhibited. The double MMP-13 protein bands are due to a better resolution of the upper proenzyme and lower active MMP-13 forms.

### IL-17-induced MMP gene expression is suppressed by mithramycin

**Figure 2:**
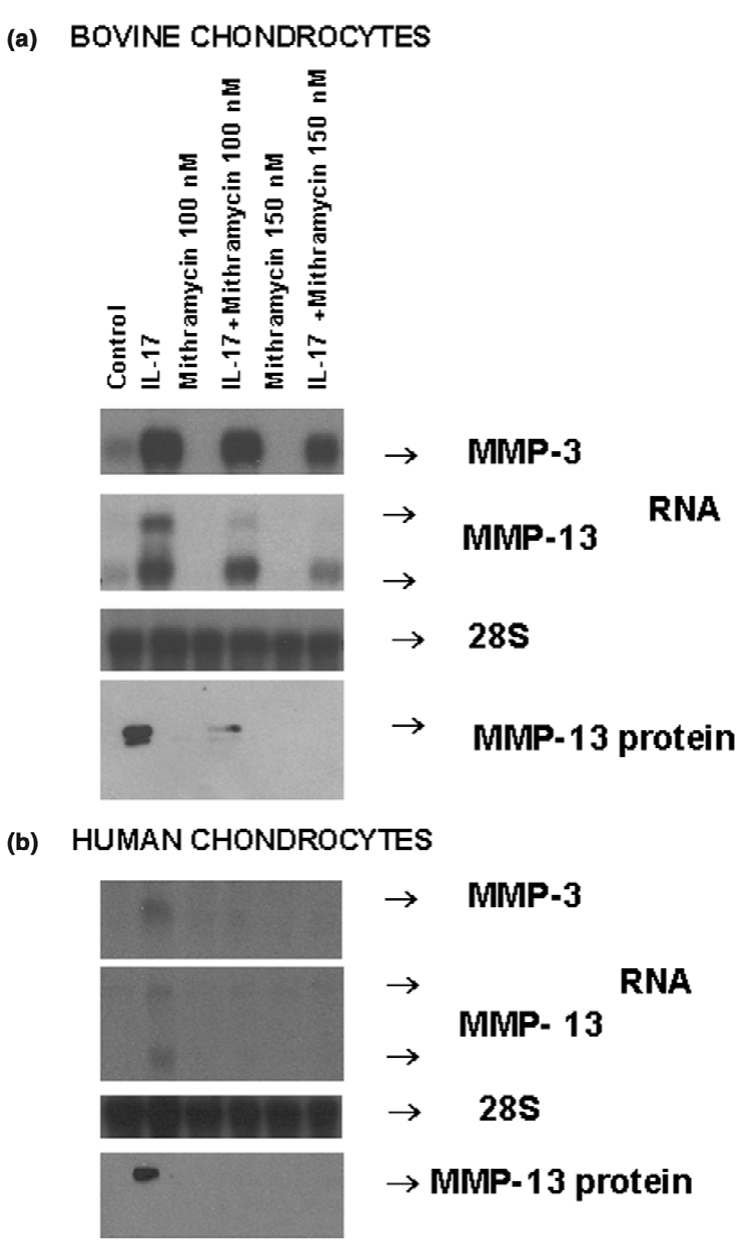
Decrease in interleukin (IL)-17-inducible matrix metalloproteinase (MMP)-3 and MMP-13 gene expression by mithramycin. Quiescent bovine chondrocytes **(a) **or primary human chondrocytes **(b) **were pretreated with different doses of mithramycin for 30 min and treated further with IL-17 for 24 hours. The MMP-3, MMP-13 and 28S RNA levels were measured by Northern hybridization, and MMP-13 protein levels were measured by Western blot analysis. For protein gels, 4 μg of protein was applied to each lane. The resulting autoradiograms indicating the respective gene products are shown.

IL-17 is a major proinflammatory cytokine and an inducer of MMP expression in chondrocytes and macrophages [11,24]. As hown in Fig. 2, IL-17 stimulated the basal MMP-3 and MMP-13 mRNA and MMP-13 protein expression in bovine and human OA chondrocytes. Mithramycin dose-dependently diminished these inductions.

### TNF-α-induced MMP gene expression is inhibited by mithramycin

**Figure 3:**
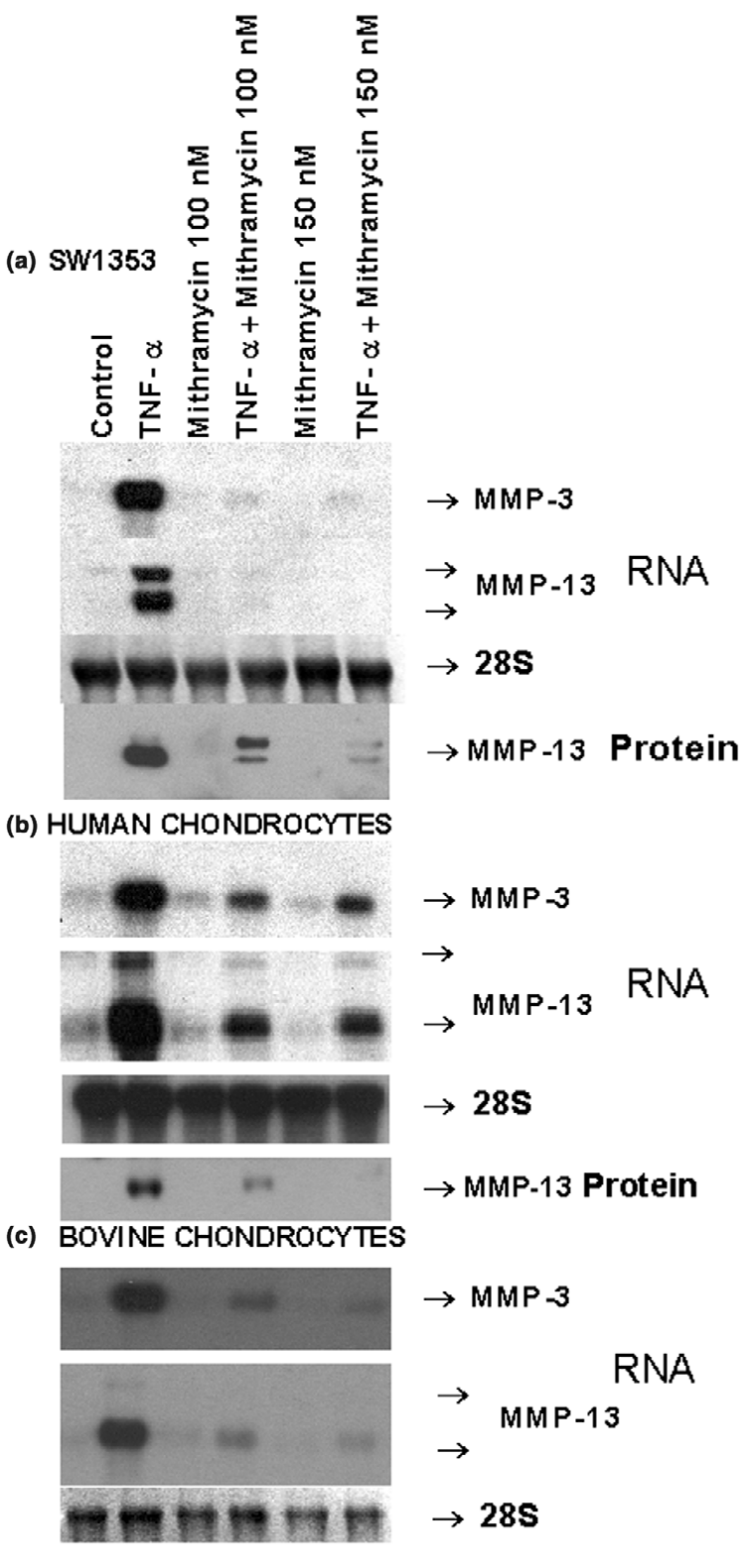
Downregulation of tumor necrosis factor (TNF)-α-inducible matrix metalloproteinase (MMP)-3 and MMP-13 RNA expression by mithramycin. Human SW1353 condrosarcoma cells **(a)**, primary human femoral head chondrocytes **(b) **and bovine chondrocytes **(c) **were pre-exposed to the indicated concentrations of mithramycin for 30 min, followed by additional treatment with TNF-α for 24 hours. The MMP-3, MMP-13 and 28S RNA levels were measured by Northern hybridization, and MMP-3 protein levels were measured by Western blot analysis. For protein gels, 3 μg **(a) **or 4 μg **(b) **of protein was applied to each lane. The resulting autoradiograms indicating the respective products are shown.

TNF-α is another prominent inflammatory cytokine that increased the constitutive MMP-3 and MMP-13 mRNA expression in chondrocytic SW1353 cells, primary human chondrocytes and bovine chondrocytes. Exposure to mithramycin followed by stimulation with TNF-α resulted in decreased constitutive and induced MMP-3 and MMP-13 gene expression (Fig. 3). In some cases, a concentration of 100 nM caused maximal inhibition; a 150 nM dose did not have any additional effect (e. g. mRNA in 3B).

### Mithramycin inhibits IL-1-stimulated expression of MMPs in human and bovine cartilage

**Figure 4:**
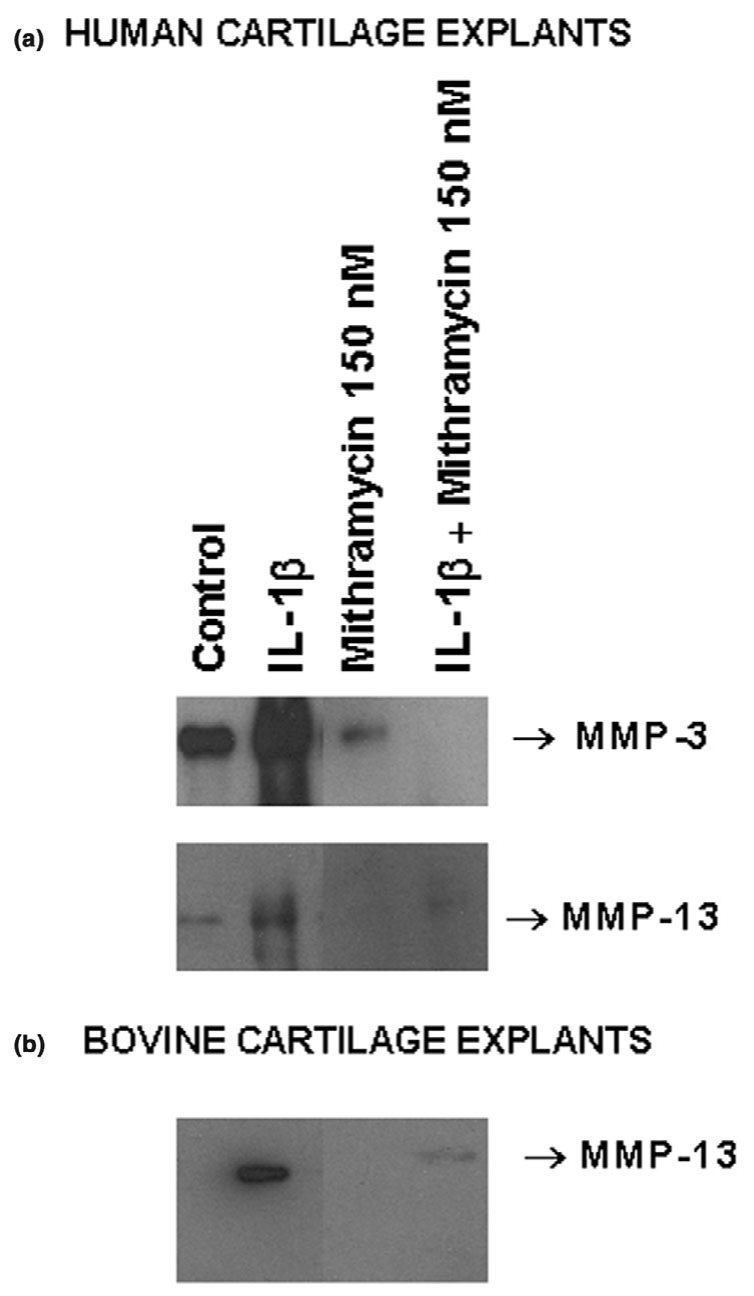
Downregulation of interleukin (IL)-1β-inducible matrix metalloproteinase (MMP) protein expression by mithramycin in cartilage explants. Human **(a) **or bovine **(b) **cartilage explants maintained in DMEM with 0.01% serum were either treated with mithramycin and IL-1β vehicles (water and PBS containing 0.1% BSA) or exposed to mithramycin (150 nM) and IL-1β (33 ng/ml) for 15 days, with renewal of the reagents every 2 days. The secreted media were concentrated by precipitation, and equal amounts of protein (human, 16 μg per lane; bovine, 20 μg per lane) were subjected to Western blotting. The MMP-3 and MMP-13 protein bands are shown.

To study the impact of mithramycin on the production of MMP-13 by chondrocytes in their native cartilage matrix, human and bovine cartilage explants were maintained in low-serum (0.01%) medium and exposed to mithramycin (150 nM) and IL-1β for 15 days with changes of reagents every 2 days. Human cartilage had somewhat elevated constitutive levels of MMP-3 and MMP-13. Mithramycin drastically reduced the secreted basal and IL-1-induced MMP-3 and MMP-13 protein levels in human cartilage (Fig. 4a) and MMP-13 in bovine cartilage (Fig. 4b) as measured by Western blotting of the conditioned media. MMP-3 levels were too low to be measurable in bovine explants. Therefore mithramycin diminishes IL-1-stimulated MMP production in cartilage explants.

### Mithramycin does not affect the phosphorylation of ERK, p38 and JNK

**Figure 5:**
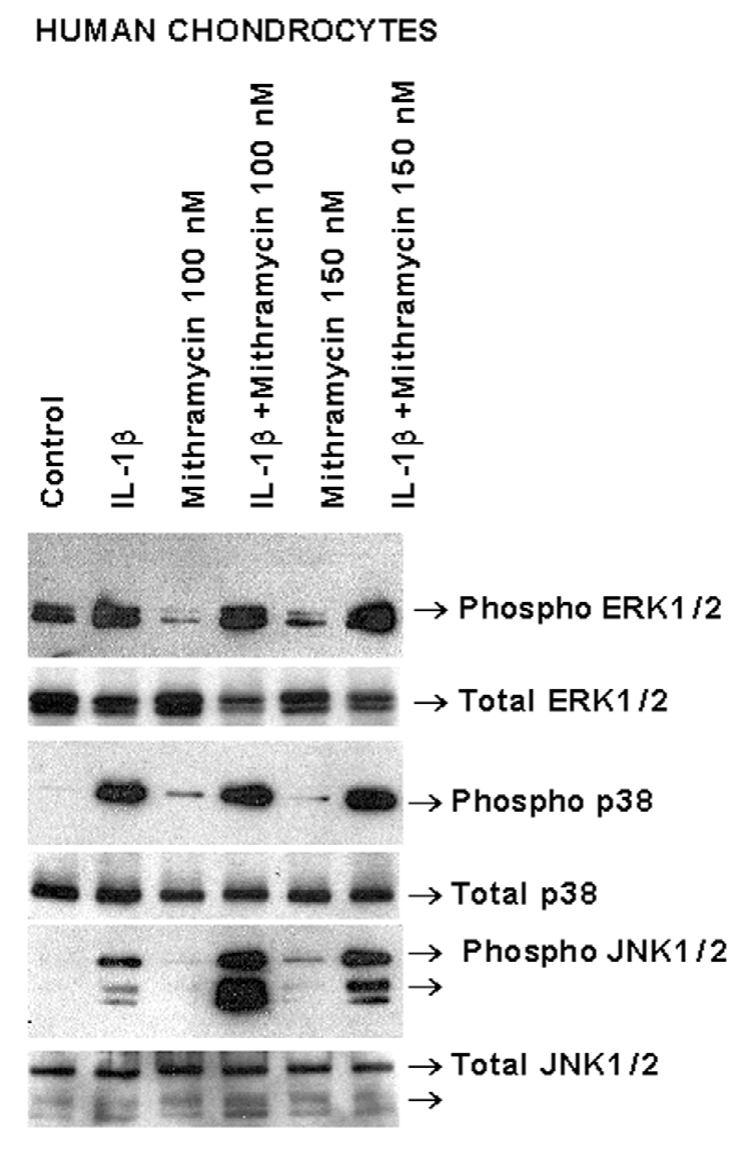
Impact of mithramycin on interleukin (IL)-1β-induced phosphorylation of mitogen-activated protein kinases. Primary human chondrocytes were pretreated with the indicated doses of mithramycin for 30 min and then stimulated with IL-1β for 20 min. Protein extracts (20 μg per lane) were analyzed by Western blotting with phosphorylation-specific and total antibodies. The resulting bands are shown. ERK, extracellular signal-regulated kinase; JNK, c-Jun N-terminal kinase.

Because MAPKs are important mediators of proinflammatory cytokine signal transduction [25], we investigated whether mithramycin affected these signaling cascades. As reported previously [25], TNF-α induced the phosphorylation of the ERK, p38 and JNK subclasses of MAPKs without affecting their total protein levels. Mithramycin did not significantly influence their phosphorylation levels (Fig. 5).

## Discussion

We have shown here that mithramycin downregulates basal and proinflammatory cytokine-stimulated MMP-3 and MMP-13 gene expression in chondrocytes and cartilage. This inhibition might be via multiple mechanisms. Sp1 is a ubiquitous transcription factor generally associated with the constitutive expression of genes. However, serum and growth-promoting conditions can stimulate its phosphorylation at specific carboxy-terminal serine residues and can affect the expression of several genes [15,20,26]. Mithramycin is a GC-specific DNA-binding drug, which prevents the binding of Sp1 to its cognate DNA [19]. MMP-3 and MMP-13 induction by the three major inflammatory cytokines and inhibition by mithramycin imply that interference with Sp1 binding might be one of the possible mechanisms. The putative Sp1 site in the MMP-13 promoter [16] might be the target of mithramycin. Because no obvious Sp1-binding site has been found in the MMP-3 promoter [13], the mechanism of MMP-3 inhibition is not known. Suppression by mithramycin might also involve indirect mechanisms. These could include blocking the transcription of other Sp1-responsive MMP regulatory genes such as *ets-1*, which has Sp1-binding sites in its promoter [27]. Analogously to our results, a requirement for Sp1 activity was demonstrated for the induction of monocyte chemoattractant protein-1 (MCP-1) by TNF-α, and a possible interaction between Sp1 and NF-κB was suggested [28]. Another possibility is that TNF-α-induced c-Jun (a component of AP-1) might superactivate Sp1, and their physical and functional interaction [29] might upregulate MMP promoters. An interaction of Sp1 and c-Jun has also been observed in the gene encoding atrial natriuretic factor [30]. ERK2 was shown to phosphorylate Sp1 [31]. IL-1 can increase the phosphorylation and activity of Sp1 in synovial fibroblasts [32]. However, in our experience, mithramycin had no effect on the IL-1-induced activation of ERK1/2, p38 and JNK MAPKs. Further, a calcium-influx-reducing agent (bis-(*o*-aminophenoxy)ethane-*N*, *N*, *N'*, *N' *-tetra-acetic acid acetoxymethyl ester (BAPTA-AM)) did not mimic the inhibition of MMP expression by mithramycin (results not shown). Thus, inhibition by mithramycin does not seem to involve MAPKs or a decrease in calcium concentration. Mithramycin might work through the aforementioned mechanisms or by interfering with Sp1/AP-1, ets-1/Sp1 and Sp1/NF-κB interactions, which are important regulators of MMPs. These hypotheses will be tested in future.

The inhibition of MMP gene expression by mithramycin is not unique to this antibiotic. Interestingly, a tetracycline analogue, doxycycline, downregulated the TNF-α-induced expression of MMP-13 RNA in human chondrocytes [33]. Similarly, tetracycline also reduced the IL-1-induced accumulation of stromelysin mRNA [34] as well as that of MMP-1 and MMP-3 in bovine chondrocytes [35]. Subsequent studies revealed that inhibition occurred by decreasing IL-1 and increasing transforming growth factor-β and its receptors, which could downregulate MMP gene expression [36]. It is not known whether mithramycin works through similar mechanisms. Mithramycin also has an interesting property of blocking bone resorption [18], which could be through the suppression of MMP gene expression. Indeed, osteoblast-derived interstitial collagenase initiates bone resorption by the generation of collagen fragments, which in turn activate bone-resorbing osteoclasts [37]. Thus, the ability of mithramycin to block the resorption of bone and cartilage (as implied here) can be advantageous in treating arthritis, in which both tissues are damaged by MMPs. Alternatively, it might work through multiple mechanisms attributed to bisphosphonates, which also prevent cartilage and bone loss and might have utility in treating arthritis [38,39]. Mithramycin is known to have several side effects in patients, including bleeding in the stomach [17], so its benefits in arthritis *in vivo *are questionable, requiring the development of safer and more specific analogues.

## Conclusion

We have shown that the upregulation of MMP-3 and MMP-13 gene expression by IL-1, IL-17 and TNF-α can be inhibited by mithramycin. The mechanisms of inhibition remain to be deciphered but do not seem to involve MAPKs. Multiple mechanisms of action similar to those of bisphosphonate may be operative. It is worth exploring whether this knowledge could lead to the development of novel therapies for blocking tissue damage in arthritis.

## Abbreviations

BSA = bovine serum albumin; DMEM = Dulbecco's modified Eagle's medium; ERK = extracellular signal-related kinase; FCS = fetal calf serum; IL = interleukin; JNK = c-Jun N-terminal kinase; MAPK = mitogen-activated protein kinase; MMP = matrix metalloproteinase; OA = osteoarthritis; PBS = phosphate-buffered saline; PCR = polymerase chain reaction; RT = reverse transcriptase; TNF = tumor necrosis factor.

## Competing interests

The author(s) declare that they have no competing interests.

## Authors' contributions

AL performed most of the tissue culture work and Western blotting experiments. JS conducted several Northern blotting and hybridization experiments. WQL cloned and tested the MMP-13 probe. MZ designed the experimental plan, coordinated the research and drafted the manuscript. All authors read and approved the final manuscript.

## References

[B1] Poole AR (1999). An introduction to the pathophysiology of osteoarthritis. Front Biosci.

[B2] Goldring MB (2000). The role of the chondrocyte in osteoarthritis. Arthritis Rheum.

[B3] Nagase H, Woessner JF (1999). Matrix metalloproteinases. J Biol Chem.

[B4] Caterson B, Flannery CR, Hughes CE, Little CB (2000). Mechanisms involved in cartilage proteoglycan catabolism. Matrix Biol.

[B5] Billinghurst RC, Dahlberg L, Ionescu M, Reiner A, Bourne R, Rorabeck C, Mitchell P, Hambor J, Diekmann O, Tschesche H (1997). Enhanced cleavage of type II collagen by collagenases in osteoarthritic articular cartilage. J Clin Invest.

[B6] Konttinen YT, Ainola M, Valleala H, Ma J, Ida H, Mandelin J, Kinne RW, Santavirta S, Sorsa T, Lopez-Otin C (1999). Analysis of 16 different matrix metalloproteinases (MMP-1 to MMP-20) in the synovial membrane: different profiles in trauma and rheumatoid arthritis. Ann Rheum Dis.

[B7] Reboul P, Pelletier JP, Tardif G, Cloutier JM, Martel-Pelletier J (1996). The new collagenase, collagenase-3, is expressed and synthesized by human chondrocytes but not by synoviocytes. A role in osteoarthritis. J Clin Invest.

[B8] Neuhold LA, Killar L, Zhao W, Sung ML, Warner L, Kulik J, Turner J, Wu W, Billinghurst C, Meijers T (2001). Postnatal expression in hyaline cartilage of constitutively active human collagenase-3 (MMP-13) induces osteoarthritis in mice. J Clin Invest.

[B9] Tetlow LC, Adlam DJ, Woolley DE (2001). Matrix metalloproteinase and proinflammatory cytokine production by chondrocytes of human osteoarthritic cartilage: associations with degenerative changes. Arthritis Rheum.

[B10] Westacott CI, Sharif M (1996). Cytokines in osteoarthritis: mediators or markers of joint destruction?. Semin Arthritis Rheum.

[B11] Shalom-Barak T, Quach J, Lotz M (1998). Interleukin-17-induced gene expression in articular chondrocytes is associated with activation of mitogen-activated protein kinases and NF-κB. J Biol Chem.

[B12] Bigg HF, Rowan AD (2001). The inhibition of metalloproteinases as a therapeutic target in rheumatoid arthritis and osteoarthritis. Curr Opin Pharmacol.

[B13] Buttice G, Quinones S, Kurkinen M (1991). The AP-1 site is required for basal expression but is not necessary for TPA-response of the human stromelysin gene. Nucleic Acids Res.

[B14] Pendas AM, Balbin M, Llano E, Jimenez MG, Lopez-Otin C (1997). Structural analysis and promoter characterization of the human collagenase-3 gene (MMP13). Genomics.

[B15] Black AR, Black JD, Azizkhan-Clifford J (2001). Sp1 and kruppel-like factor family of transcription factors in cell growth regulation and cancer. J Cell Physiol.

[B16] Wu N, Opalenik S, Liu J, Jansen ED, Giro MG, Davidson JM (2002). Real-time visualization of MMP-13 promoter activity in transgenic mice. Matrix Biol.

[B17] Zojer N, Keck AV, Pecherstorfer M (1999). Comparative tolerability of drug therapies for hypercalcaemia of malignancy. Drug Saf.

[B18] Kiang DT (1978). Effect of mithramycin on bone beta-glucuronidase and resorption. Calcif Tissue Res.

[B19] Blume SW, Snyder RC, Ray R, Thomas S, Koller CA, Miller DM (1991). Mithramycin inhibits SP1 binding and selectively inhibits transcriptional activity of the dihydrofolate reductase gene in vitro and in vivo. J Clin Invest.

[B20] Ihn H, Tamaki K (2000). Increased phosphorylation of transcription factor Sp1 in scleroderma fibroblasts: association with increased expression of the type I collagen gene. Arthritis Rheum.

[B21] Chomczynski P, Sacchi N (1987). Single-step method of RNA isolation by acid guanidinium thiocyanate-phenol-chloroform extraction. Anal Biochem.

[B22] Shlopov BV, Lie WR, Mainardi CL, Cole AA, Chubinskaya S, Hasty KA (1997). Osteoarthritic lesions: involvement of three different collagenases. Arthritis Rheum.

[B23] Freije JM, Diez-Itza I, Balbin M, Sanchez LM, Blasco R, Tolivia J, Lopez-Otin C (1994). Molecular cloning and expression of collagenase-3, a novel human matrix metalloproteinase produced by breast carcinomas. J Biol Chem.

[B24] Jovanovic DV, Martel-Pelletier J, Di Battista JA, Mineau F, Jolicoeur FC, Benderdour M, Pelletier JP (2000). Stimulation of 92-kd gelatinase (matrix metalloproteinase 9) production by interleukin-17 in human monocyte/macrophages: a possible role in rheumatoid arthritis. Arthritis Rheum.

[B25] Liacini A, Sylvester J, Li WQ, Huang W, Dehnade F, Ahmad M, Zafarullah M (2003). Induction of matrix metalloproteinase-13 gene expression by TNF-α is mediated by MAP kinases, AP-1, and NF-κB transcription factors in articular chondrocytes. Exp Cell Res.

[B26] Black AR, Jensen D, Lin SY, Azizkhan JC (1999). Growth/cell cycle regulation of Sp1 phosphorylation. J Biol Chem.

[B27] Oka T, Rairkar A, Chen JH (1991). Structural and functional analysis of the regulatory sequences of the ets-1 gene. Oncogene.

[B28] Ping D, Boekhoudt G, Zhang F, Morris A, Philipsen S, Warren ST, Boss JM (2000). Sp1binding is critical for promoter assembly and activation of the MCP-1 gene by tumor necrosis factor. J Biol Chem.

[B29] Kardassis D, Papakosta P, Pardali K, Moustakas A (1999). c-Jun transactivates the promoter of the human p21(WAF1/Cip1) gene by acting as a superactivator of the ubiquitous transcription factor Sp1. J Biol Chem.

[B30] McDonough PM, Hanford DS, Sprenkle AB, Mellon NR, Glembotski CC (1997). Collaborative roles for c-Jun N-terminal kinase, c-Jun, serum response factor, and Sp1 in calcium-regulated myocardial gene expression. J Biol Chem.

[B31] Merchant JL, Du M, Todisco A (1999). Sp1 phosphorylation by Erk 2 stimulates DNA binding. Biochem Biophys Res Commun.

[B32] Ray A, Schatten H, Ray BK (1999). Activation of Sp1 and its functional co-operation with serum amyloid A-activating sequence binding factor in synoviocyte cells trigger synergistic action of interleukin-1 and interleukin-6 in serum amyloid A gene expression. J Biol Chem.

[B33] Shlopov BV, Smith GN, Cole AA, Hasty KA (1999). Differential patterns of response to doxycycline and transforming growth factor β1 in the down-regulation of collagenases in osteoarthritic and normal human chondrocytes. Arthritis Rheum.

[B34] Jonat C, Chung FZ, Baragi VM (1996). Transcriptional downregulation of stromelysin by tetracycline. J Cell Biochem.

[B35] Sadowski T, Steinmeyer J (2001). Effects of tetracyclines on the production of matrix metalloproteinases and plasminogen activators as well as of their natural inhibitors, tissue inhibitor of metalloproteinases-1 and plasminogen activator inhibitor-1. Inflamm Res.

[B36] Shlopov BV, Stuart JM, Gumanovskaya ML, Hasty KA (2001). Regulation of cartilage collagenase by doxycycline. J Rheumatol.

[B37] Holliday LS, Welgus HG, Fliszar CJ, Veith GM, Jeffrey JJ, Gluck SL (1997). Initiation of osteoclast bone resorption by interstitial collagenase. J Biol Chem.

[B38] Catterall JB, Cawston TE (2003). Drugs in development: bisphosphonates and metalloproteinase inhibitors. Arthritis Res Ther.

[B39] Hayami T, Pickarski M, Wesolowski GA, McLane J, Bone A, Destefano J, Rodan GA, Duong le T (2004). The role of subchondral bone remodeling in osteoarthritis: reduction of cartilage degeneration and prevention of osteophyte formation by alendronate in the rat anterior cruciate ligament transection model. Arthritis Rheum.

